# Neoantigen Presentation and IFNγ Signaling on the Same Tumor-associated Macrophage are Necessary for CD4 T Cell–mediated Antitumor Activity in Mice

**DOI:** 10.1158/2767-9764.CRC-22-0052

**Published:** 2022-05-09

**Authors:** Ainhoa Perez-Diez, Xiangdong Liu, Polly Matzinger

**Affiliations:** Ghost Lab, T Cell Memory and Tolerance Section, Laboratory of Cellular and Molecular Immunology, National Institutes of Allergy and Infectious Diseases, NIH, Bethesda, Maryland.

## Abstract

Tumor-associated macrophages (TAM) promote tumor survival, angiogenesis, and metastases. Although they express MHC class II molecules, little is known about their ability to present tumor antigens to tumor-infiltrating CD4 T cells, and the consequences of such presentation. To answer these questions, we used a C57/BL10 mouse tumor model where we subcutaneously implant a bladder carcinoma cell line naturally expressing the H-Y male antigen into female mice, making the H-Y antigen a *de facto* neoantigen. We found that TAMs indeed present tumor antigens to effector CD4 T cells and that such presentation is necessary for tumor rejection. As a consequence of this interaction, TAMs are reeducated to produce lower amounts of tumor-promoting proteins and greater amounts of inflammatory proteins. The reeducation process of the TAMs is transcriptionally characterized by an IFNγ signature, including genes of known antiviral and antibacterial functions. CD4 production of IFNγ, and not TNFα or CD40L, is required for the reeducation process and tumor rejection. Furthermore, IFNγ signaling on antigen-presenting TAMs and not on bystander TAMs, is necessary for the antitumor effect. These data identify critical mechanisms of tumor rejection by CD4 T cells and underscores the importance of effector CD4 T cell–tissue macrophage interactions not only at the tumors site but potentially in other tissues.

**Significance:**

In the tumor microenvironment, TAMs are capable of presenting tumor antigens to effector CD4 T cells. Upon antigen recognition, the CD4 cells transform transcriptionally, phenotypically, and functionally the TAMs inducing tumor rejection. This reeducation process requires both cognate interaction and IFNγ signaling on the same macrophage.

## Introduction

We have previously found that several different tumors that are resistant to clearance by CD8 T cells could be cleared by tumor-specific CD4 T cells, in the absence of any other T cell; and that to achieve tumor rejection, the antitumor CD4 T cells did not need to directly recognize the tumor cells, but rather partnered with a local stromal cell type (1). In addition, more recent clinical trials have shown that CD4 T cells specific against mutated neoantigens can successfully be used alone or in combination to checkpoint inhibitors to treat patients with cancer (2–4). However, the mechanism of CD4-mediated tumor rejection has not been clearly delineated.

Macrophages are the most abundant immune cell population found in both human and mouse solid tumors during all phases of tumor development. These macrophages, called tumor-associated macrophages (TAM), have been extensively studied for their important roles in supporting tumor origination, angiogenesis, and metastasis formation, as well as for their role in the generation of an immunosuppressive tumor environment (5). TAMs display similar functional characteristics as M2 macrophages (6), such as the production of tumor-promoting factors as the angiogenic molecule, VEGF; chemokines such as CCL2 (MCP-1) and CCL22 (MDC); cytokines like IL10, which can suppress Th1 tumor–rejecting immune responses; metalloproteinases like MMP-9, which helps tumor cells to navigate through the extracellular matrix to develop metastases (5); and MFG-E8, which promotes resistance of cancer stem cells to antitumor drugs (7). In contrast, TAMs do not produce significant amounts of inflammatory products characteristic of classically activated, or M1, macrophages, such as CXCL-9 (MIG), IL1α, TNFα, RANTES (CCL5; ref. 5), and Activin A (8). Not surprisingly, high number of TAMs has been associated with poor cancer prognosis (9). Hence, several experimental antitumor therapies have attempted to eliminate TAMs (10), or to inhibit their precursors migration to the tumor site (11). Alternatively, the presence of TAMs with M1 phenotype, when found, has been correlated with better clinical outcome (12), suggesting that not only TAMs numbers but also their polarization status, M2 versus M1, is relevant for tumor prognosis. Thus, another potential therapeutic strategy is to reverse their phenotype toward M1 macrophages. Previous studies have shown that it is possible to obtain a partial phenotype reversal in *ex vivo* purified TAMs by delivering IL12 intratumorally (13), by stimulating with anti-CD40 antibodies (14) or by treatment with a class IIa histone deacetylase inhibitor (15).

Even though TAMs have been shown to express MHC class II molecules in both clinical samples and mouse models (16) and that macrophages have been known for a long time to be important APCs for immune responses (17), there is little information on their potential function as antigen-presenting cells (APC) of tumor antigens to CD4 T cells. We wondered, therefore, whether tumor-specific CD4 T cells might interact with local TAMs, and reeducate them *in vivo* to overcome local tumor-derived signals.

At least two features suggested that the TAM phenotype might not be unalterably fixed but receptive to the influence of an appropriate CD4 T cell. First, an important characteristic of both human and mouse macrophages is their plasticity in response to typical CD4 products. *In vitro* studies have shown, for example, that INFγ stimulation skews macrophages toward the inflammatory M1 phenotype, whereas IL4 and/or IL13 stimulation skews macrophages toward the “alternatively activated” or M2 phenotype (18). Second, CD4 T cells can induce a functional switch in many immune cells. Th1 CD4 T cells can activate macrophages to effectively kill intracellular bacteria (19). They also can “license” dendritic cells (DC) to stimulate effective CTL (19), and to produce copious amounts of IL12 (20). Orally immunized CD4 T cells can educate DCs to induce naïve T cells to become Th2/3 effectors (21). And follicular helper CD4 T cells direct the class of antibodies made by B cells (22). In all these cases, the CD4 T cells modulatory/helper activity is antigen specific and the CD4 T cell needs to recognize its antigen presented by the MHC class II molecule of the modulated/helped cell (macrophages, DC, or B cells).

To test whether TAMs can present tumor antigens on MHC class II to effector CD4 T cells at the tumor site and the consequences of such interaction, we used a previously described mouse model where Marilyn CD4 T cells, specific for the male antigen H-Y, is very efficient rejecting H-Y–expressing tumors (1). We found that not only TAMs could indeed present tumor antigens to tumor-infiltrating CD4 T cells, but that this presentation was required for tumor rejection. The CD4 T cells induced a transcriptional and functional switch in the TAMs, converting them from tumor-nurturing macrophages to inflammatory macrophages. The reeducation of the TAMs required IFNγ but not CD40L. Furthermore, both antigen presentation and IFNγ signaling needed to converge on the same macrophage for optimal antitumor effect.

## Materials and Methods

### Cell Lines

The H-2^b^ bladder carcinoma MB49 cell line, a gift from Dr. Fraia Melchionda (NCI, NIH) in 2002, was cultured in complete Iscove's Modified Dulbecco's Medium (IMDM; IMDM plus 10% FCS, glutamine, and antibiotics) and tested for *Mycoplasma* and other mouse pathogens by the IMPACT (Infectious Microbe PCR AmplifiCation Test) before starting mouse experimentation. Subsequently, cells were retested every 2 years for *Mycoplasma* using the MycoAlert Detection Kit (Lonza) and always found to be negative. Cells were cultured from 2 to 6 days before mouse challenge and were discharged if the culture had been stressed. Cells authentication was not conducted.

### Mice, Tumor Challenge, and Treatment

The anti-H-Y T-cell receptor (TCR) transgenic mice Marilyn, and A1M have been described previously (23, 24). They, C57BL/10, C57BL/10-RAGKO, and B10.A-RAGKO mice were obtained from Taconic Farms. MarilynIFNγKO cells were a gift from Mathew Albert (Institut Pasteur). RAGKO-IFN-γRKO (RAGIFNγRdKO) mice were generated by crossing C57BL/10-RAGKO with C57BL/6-IFNγRKO (Jackson Laboratory) and then intercrossing to homozygosity. Only female mice were used and they were housed in specific pathogen-free conditions. All animal handling and experiments were conducted under protocols approved by the NIAID Institutional Animal Care and Use Committee. The NIH is an Association for Assessment and Accreditation of Laboratory Animal Care International–accredited facility.

**FIGURE 1:**
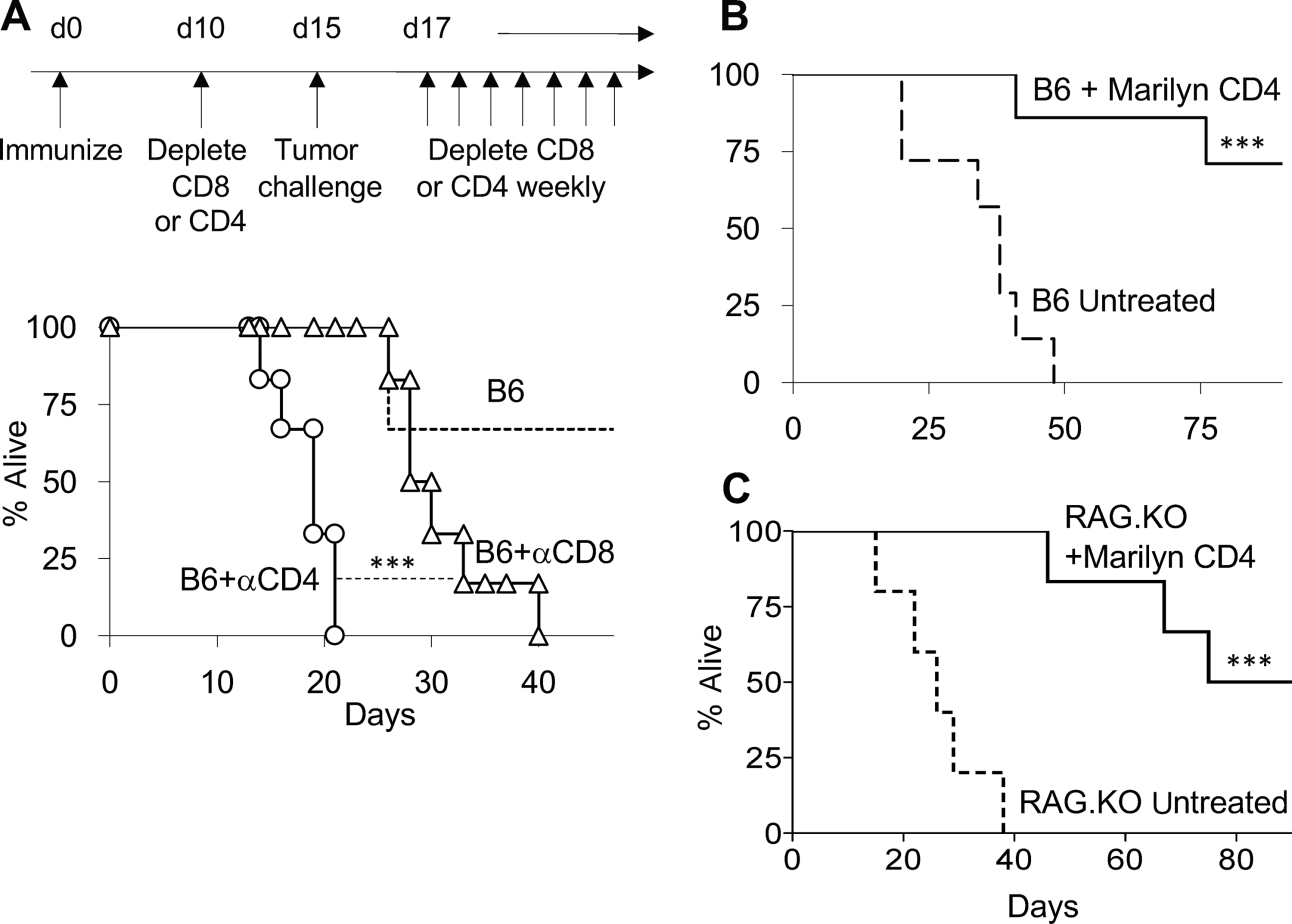
Tumor-specific CD4 T cells have antitumor effector properties beyond helping CD8 T cells. **A,** Experimental timeline, above, and survival graph below. Wild-type female mice were immunized with male splenocytes. Ten days later, mice were depleted of either CD4 or CD8 T cells (circles and triangles, respectively), or were not depleted (dotted line). Five days after depletion, all mice received H-Y–expressing MB49 tumor cells. Depleting antibodies were given during the length of the experiment. Wild-type (**B**) or RAG.KO (**C**) female mice received MB49 tumor. One day later, mice in each group either received Marilyn CD4 T cells (continuous line) or were left untreated (discontinuous line). Mice survival was followed until all remaining mice were tumor free. All the experiments were done at least two times.***, *P* < 0.001 using log-rank test.

After thawing, 10^5^ MB49 cells in 100 μL of PBS were injected subcutaneously into the mice flank. One to 5 days later, mice received 10^6^ cells of freshly isolated spleen and mesenteric lymph nodes from the TCR transgenic mice. Tumor size was measured every 3–4 days and the volume calculated as length × width × height/2. Mice were sacrificed if they became distressed or if tumor volume became >1 cm^3^. In Fig. 1A, C57BL/10 mice were immunized with 2 × 10^6^ male splenocytes intraperitoneally. For depletion of CD4 T cells, a combination of GK1.5 (500 μg) and YTA 3.1.2. (100 μg) anti-CD4 depleting antibodies was given at days 0, 3, and every 7 days thereafter. To deplete CD8 T cells, anti-CD8 depleting antibody YTS 169.4 (100 μg) was given every 7 days.

### Flow Cytometry and Cell Sorting

Tumors were dissected from mice, disaggregated without the use of digestion enzymes, and passed through a filter. After blocking nonspecific binding with ultrablock solution (a 1:1:1 mixture of rat, hamster, and mouse sera, with 10 μg/mL 2.4G2 mAb), cells were stained with various combinations of antibodies (for specific antibodies, see Supplementary Experimental Procedures). Dead cells were excluded by staining with 7AAD (BD Pharmingen). Flow cytometry was performed on a FACSCalibur or FACSCanto (BD Biosciences). Analysis of flow cytometry data was done using Cell Quest (BD Biosciences) or Flowjo (Treestar). Cell sorting was performed using a BD FACSAria II (BD Biosciences). TAMs were sorted on the basis of the markers CD45^+^, CD11b^+^, MHC ClassII^+^, Gr-1^neg^, and 7AAD^neg^. CD4 T cells were sorted on the basis of the markers CD45^+^, CD4^+^, TCR^+^, and 7AAD^neg^.

### *In Vitro* Stimulation of CD4 T Cells with TAMs

To test tumor antigen presentation capabilities of bone marrow–derived cells at the tumor site, three populations of CD11b^+^/CD45^+^/7AAD^neg^ cells were sorted; population #I, class II^+^/Gr-1^neg^; #II, class II^neg^/Gr-1^neg^; and #III, class II^neg^/Gr-1^+^. A total of 4 × 10^4^ cells from each population were cultured overnight together with a mixture of 5 × 10^4^ sorted naive Marilyn CD4 cells plus 5 × 10^4^ A1M CD4 cells, in 200 μL of complete IMDM (plus 50 μmol/L β−mercaptoethanol) in 96-well plates. As negative controls, each of the sorted populations was plated by itself. As a positive control for maximal antigen presentation, TAM population #I was cultured with the sorted Marilyn CD4 TCR transgenic cells with the addition of 5 mmol/L of Dby peptide, the cognate epitope of Marilyn CD4 T cells.

To test cytokine production by the CD4 tumor-infiltrating lymphocytes (CD4 TIL), we sorted the CD4 T cells present at the tumor site 7 days after CD4 T-cell transfer, plated 1.5 × 10^4^ CD4 T cells in 200 μL of complete IMDM in 96-well plates, and stimulated them 2 ways. First, with 1.5 × 10^4^ sorted class II^+^ TAMs (population #I) from untreated mice that had received tumor cells 12 days earlier. Second, with plate-bound anti-CD3 (0.2 μg/mL; 2C11, BD Biosciences) and anti-CD28 (14 μg/mL; ascites) antibodies. Supernatants were collected 24 hours later and assessed for cytokines using the Quantibody Mouse Interleukin Array 1 (Raybiotech, Inc.).

### Bone Marrow Chimeras

For bone marrow chimera experiments, host mice were irradiated with 900 rads and reconstituted the same day with bone marrow cells from different donors. Successful donor reconstitution was checked by flow cytometry of blood cells, staining for MHC Class I molecules at 6–8 weeks after irradiation. One to 2 weeks later, mice were injected with tumor cells, and then given Marilyn CD4 T cells a day later. In the experiment shown in Fig. 3, all mice were immunized with 2 × 10^6^ male splenocytes intraperitoneally at the day of CD4 T-cell transfer and every 7 days thereafter for the entire length of the experiment to ensure adequate priming of the CD4 T cells.

### Phenotypical, Functional, and Molecular Comparison of Untreated Versus *In Vivo* CD4-Treated TAMs

For experiments comparing TAMs from tumor-bearing RAGKO mice that were either untreated (untreated TAMs) or CD4-treated (CD4-TAMs), mice were first challenged with 10^5^ MB49 tumor cells subcutaneously. Five days later, half of the mice received Marilyn CD4 T cells intraperitoneally. All mice were euthanized 12 days after tumor challenge. The tumors were dissociated as described above, and cells stained with different antibodies to compare either the phenotype of untreated versus CD4-treated TAMs or their protein production. For the latter, between 10^5^ and 1.25 × 10^5^ sorted TAMs were plated in 96-well plates with 200 μL of complete IMDM. TAMs came from either individual mice or from mice pooled within the same experiment (to be able to reach at least 10^5^ TAMs per assay). Supernatants were collected 18 hours later and send to Raybiotech, Inc. to measure cytokines and chemokines characteristic of M2 or M1 macrophages using a custom Cytokine array (see panel of cytokines tested in Fig. 4). Amount of protein in the supernatant was normalized per 10^5^ cells. For the microarray experiments, sorted TAMs were lysed immediately post sort for RNA isolation using miRNeasy mini kit (Qiagen). RNA samples were labeled, hybridized, and arrays were scanned as described below.

### *In Vivo* Blocking of Cytokines and CD40L

To block IFNγ, TNFα, or CD40L, 500 μg of anti-IFNγ antibody (clone XMG1.2) or of anti-TNFα antibody (clone XT3.11), or 800 μg of anti-CD40 L antibody (clone MR1) were injected intraperitoneally at days 2, 5, and 7 after CD4 T-cell transfer, and every 7 days thereafter. The same amount and schedule of rat (for the cytokines) or hamster (for CD40L) IgG1 antibody was injected in the control group of mice. All antibodies were purchased from BioXCell.

### Sample Preparation and Hybridization for Microarray Experiments

RNA quality was verified by Bioanalyzer with RNA integrity number greater than 8.5 for all samples. Microarray target material was prepared by amplification of 50 ng total RNA using NuGEN WT-Ovation systems V2, followed by biotinylation with NuGEN Encore BiotinIL, using 4 μg input of cDNA. We applied 2 μg of conjugate to Illumina Mouse WG-6 v2.0 Expression BeadChip microarrays and hybridized at 48°C for 16 hours, postprocessed per manufacturer's instructions and scanned using a HiScan (Illumina). Images were analyzed using Genome Studio software (Illumina) and tabular data uploaded to the mAdb database (http://mAdb.niaid.nih.gov).

### Analysis of Microarray Data

Microarray data were analyzed using BRB Array Tools developed by the Biometric Research Branch of the NCI (http://linus.nci.nih.gov/BRB-ArrayTools.html). Array data were filtered to threshold the spot intensity of the probes at the minimum value if the spot intensity was <90, and a quantile normalization was applied. For nonsupervised analysis, hierarchical clustering of the samples, centering the genes using centered correlation and average linkage was done using 10 samples (five untreated TAMs and five CD4-treated TAMs with one technical replicate in each group). For class comparison analysis, differentially expressed genes between the two groups of samples were identified by random variance *t*-test setting the *P* value at <0.001.

### Statistical Analysis

Two-tailed Mann–Whitney *t* test was used when comparing two groups of samples. For comparison of three or more groups, one-way ANOVA Kruskal–Wallis with Dunn as posttest was applied. Log-rank test was applied on survival graphs.

### Data Availability

The data generated in this study are available within the article and its Supplementary Data files. The full macrophage transcriptomic data is publicly available in Gene Expression Omnibus at GSE40920.

## Results

### Tumor-Specific CD4 T Cells have Antitumor Effector Properties Beyond Helping CD8 T Cells

To test the role of antitumor CD4 T effector cells as part of a natural endogenous response, we depleted female C57Bl/6 (B6) mice of their CD4 or CD8 T cells 5 days before challenging them with the bladder carcinoma MB49 cell line, which spontaneously expresses the H-Y male antigen. Mice were immunized against the H-Y antigen 10 days before the depletion to ensure that the CD8 T cells received proper help from CD4 T cells during the priming phase (see timeline on Fig. 1A). CD4 depleted mice lost most of the antitumor response, surviving barely 20 days, while CD8 depleted mice survived significantly longer (Fig. 1A), suggesting that CD4 T cells may have an additional role in tumor rejection besides helping CD8 T cells. To test the CD4-mediated antitumor effect, we transferred CD4 T cells from the Marilyn anti H-Y TCR transgenic mouse (which has no CD8 T cells) into nonimmunized tumor-bearing B6 mice. Most of the mice receiving Marilyn CD4 T cells survived while all the untreated mice died within 50 days (Fig. 1B). These two experiments show that both vaccine-elicited endogenous CD4 T cells as well as transferred TCR transgenic CD4 T cells are necessary for tumor rejection in immune-competent mice. The depletion experiment suggests that there is a mechanism of CD4-mediated antitumor effect independent of helping CD8 T cells. To further characterize such a mechanism without any confounding CD8-mediated effects, we next moved to a model that totally lacked CD8 T cells. For this, we transferred Marilyn CD4 T cells into tumor-bearing RAG.KO mice, which has no endogenous CD8 T cells, and found that 50% of the mice rejected the tumor and all of them had a survival advantage respect to untreated mice (Fig. 1C; ref. 1). These data show that CD4 T cells can reject tumors in absence of CD8 T cells.

### TAMs Present Tumor Antigens to CD4 T Cells at the Tumor Site

**FIGURE 2:**
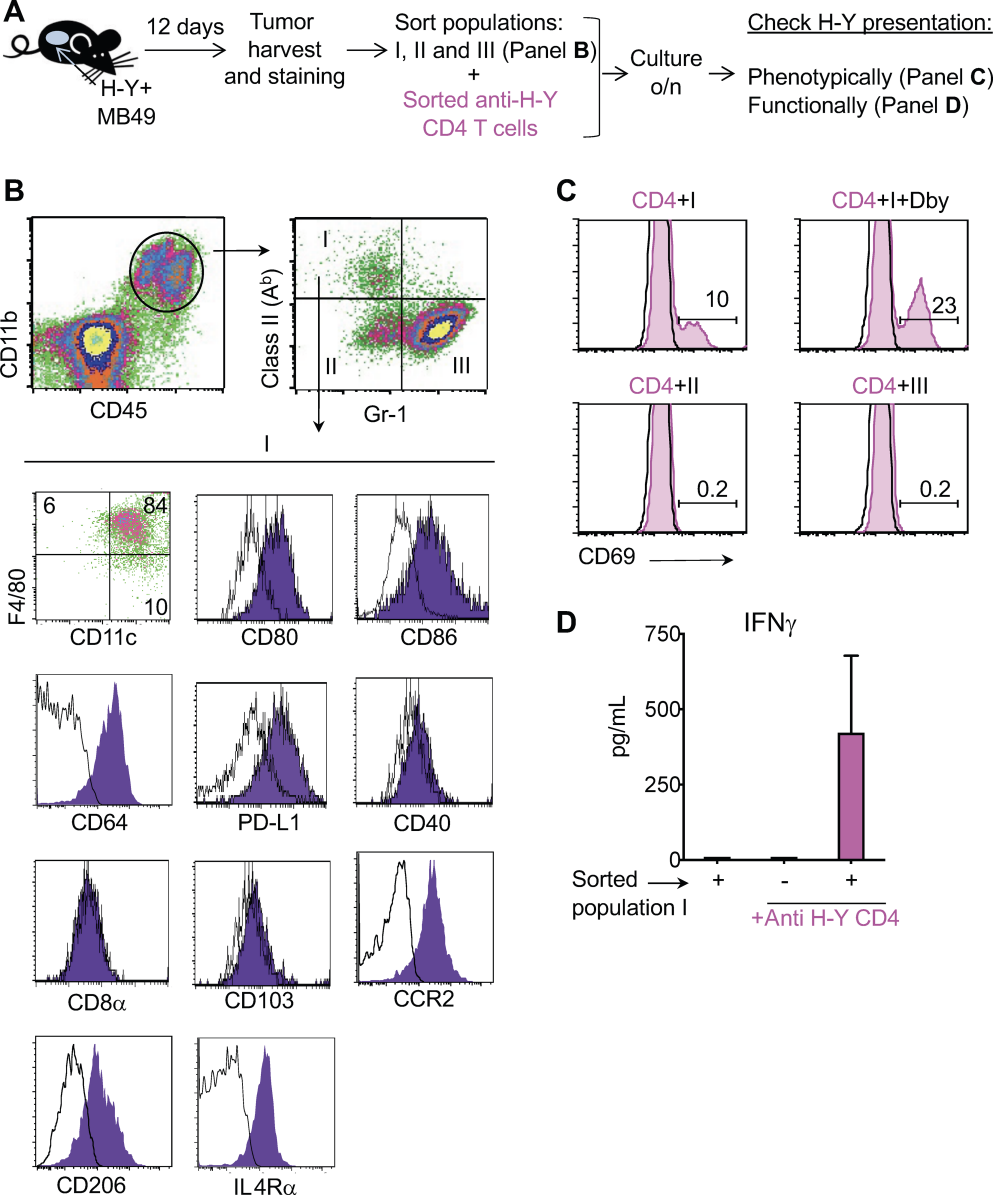
TAMs present tumor antigens to CD4 T cells at the tumor site. **A,** Schematic representation of the experiment. **B,** H-Y–expressing MB49 tumor growing subcutaneously in B10.RAGKO mice was mechanically dissociated at day 12 after tumor challenge and analyzed by flow cytometry. Live cells were gated using 7AAD and hematopoietic cells of myeloid origin were identified as CD45.2^+^ and CD11b^+^. CD45.2^+^/CD11b^+^ cells were divided into three populations based on their expression of MHC class II (A^b^) and Gr-1 (population I, class II^+^ /Gr-1^neg^; II, class II^neg^/Gr-1^neg^; and III, class II^neg^/Gr-1^+^). Population I was further phenotypically characterized by looking at F4/80, CD11c, CD80, CD86, CD64, PD-L1, CD40, CD8α, CD103, CCR2, CD206, and IL4Rα expression. A representative experiment from 10 is shown. **C,** Each of the three populations described in **B** were sorted and used as stimulators *in vitro* for naïve sorted anti-H-Y Marilyn (A^b^ restricted) and A1M (A^k^ restricted) CD4 T cells. Population I was also cultured in presence of 5 mmol/L of the H-Y peptide Dby. Twenty-four hours later, the cultures were harvested, and CD69 expression was analyzed on Marilyn cells (gated as CD4^+^/TCR^+^/Vb6^+^ T cells, pink shading), and A1M cells (gated as CD4^+^/TCR^+^/Vb6^neg^, black line). A representative experiment of three is shown. **D,** Population I was used to stimulate sorted *in vivo* primed Marilyn CD4 T cells, and IFNγ was measured in the supernatant 24 hours later. The average of two experiments with the SD is shown.

In our previous study, we found that CD4 T cells can reject tumors that do not express MHC class II molecules (1), suggesting that an MHC class II+ host cell may capture and present the tumor antigen to CD4 T cells at the tumor site. We next set up to identify the class II+ cell at the tumor site responsible for tumor antigen presentation to CD4 T cells. For this, we injected MB49 into RAGKO mice, 12 days later harvested the tumors along with their infiltrating stromal cells and used flow cytometry to identify and characterize the CD11b^+^ MHC class II+ myeloid cells (Fig. 2A and B). Figure 2B shows that the CD11b^+^/class II^+^ cells were Gr1^neg^ and positive for both F4/80, a macrophage marker, and CD11c, a DCs marker. They also expressed CD80, CD86, CD64, and PD-L1, very low levels of CD40, and were negative for CD8α and CD103. This combination of surface markers resembles a type of cell that has previously been identified as both a DC (25–28) and a macrophage (29) and it is found in tissues, rather than in secondary lymphoid organs. These cells also expressed TAM and M2 macrophage markers such as CCR2, the Mannose receptor (CD206), and IL4Rα (CD124) (Fig. 2G). To test whether these TAMs can capture *in vivo* tumor antigens and present them to CD4 T cells, we sorted three populations of tumor-infiltrating myeloid CD11b^+^ cells based on Gr-1 and class II expression (Fig. 2B) and used them to stimulate naïve, sort-purified, Marilyn CD4 T cells overnight without the addition of antigen (Fig. 2A). To measure functional antigen presentation, we monitored the expression of the T-cell surface marker, CD69, as naïve CD4 T cells are CD69 negative but rapidly become positive upon antigen recognition. We saw that the Marilyn CD4 T cells upregulated CD69 expression when cultured with the TAMs (population I, class II+/ Gr-1^neg^ cells; Fig. 2C), indicating that the TAMs had captured the H-Y antigen *in vivo* from the tumor and were able to present it to stimulate the Marilyn CD4 T cells. In contrast, neither population II (Gr-1^neg^/Class II^neg^), nor population III (Gr-1^+^ cells), were able to stimulate the Marilyn CD4 T cells. To control for the possibility that CD69 upregulation might be triggered by the release of stimulatory cytokines by the TAMs during the overnight *in vitro* culture, rather than by antigen presentation, we added a second CD4 TCR transgenic cell (A1M) to the cultures (Fig. 2C, black line) that cannot recognize H-Y antigen in this setting. The upregulation of CD69 by Marilyn CD4 T cells, but not by A1M CD4 T cells, shows that the stimulatory property of population I was antigen specific. As positive control of the assay, we externally added 5 mmol/L of Dby (Maryland cognate peptide) to the culture of population I plus the CD4 T cells. We found that population I had captured enough tumor antigen *in vivo* to present at 50% of the maximum efficiency found when the peptide was externally added (Fig. 2C, compare top left and right histograms). The antigen presentation was not due to contaminating APCs in the sorted T-cell population (Supplementary Fig. S1). Sorted TAMs (population I) were also able to induce the production of IFNγ by previously primed Marilyn CD4 T cells without the external addition of antigen (Fig. 2D). Altogether, these data show that TAMs capture and present tumor antigens to CD4 T cells at the tumor site, inducing IFNγ production by antigen specific effector T cells.

### Presentation of Tumor Antigen by Myeloid Cells is Necessary for Tumor Rejection by Effector CD4 T Cells

**FIGURE 3:**
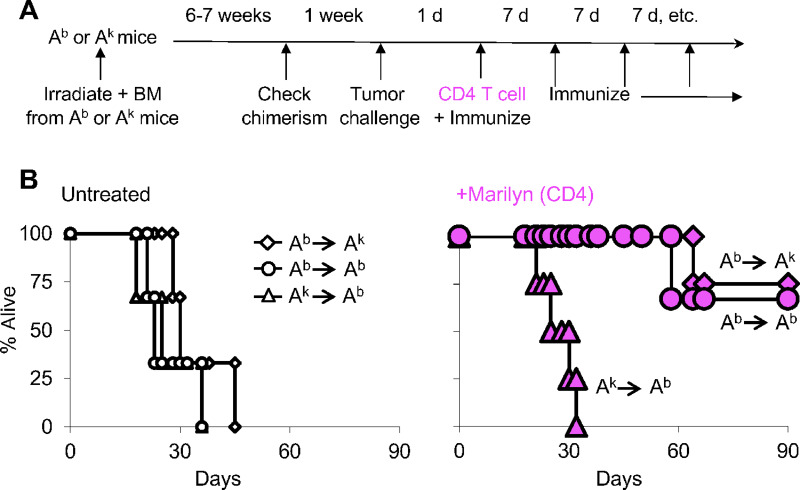
Presentation of tumor antigen by myeloid cells is necessary for tumor rejection by effector CD4 T cells. **A,** Schematic representation of the bone marrow chimera experiment. We made bone marrow chimeras in which the bone marrow–derived cells expressed the right (A^b^, circles) or wrong (A^k^, triangles) MHC class II allele to present the tumor antigen to Marilyn CD4 T cells. This was accomplished by using B10 (A^b^) or B10.A (A^k^) RAG.KO strain of mice. As additional control group, we also transferred A^b^ bone marrow into A^k^ mice (diamonds). Tumor-bearing mice were immunized with male A^b^ splenocytes to ensure efficient priming of the CD4 T cells. **B,** Survival of tumor-bearing mice was followed in the three different groups of chimera mice that either were left untreated or treated with Marilyn CD4 T cells, left and right panels, respectively.

Next, we evaluated the role that tumor antigen presentation by bone marrow–derived myeloid cells to CD4 T cells might play on antitumor effect. For this, we transferred bone marrow cells from donors whose class II molecules have the restriction element able (A^b^) or not (A^k^) to present H-Y antigen to Marilyn T cells. When chimerism was established, we challenged the mice with tumor and transferred CD4 T cells. At this time, mice were immunized with irradiated A^b^ male splenocytes (expressing H-Y antigen) and every 7 days thereafter (Fig. 3A). This ensured that CD4 T cells were efficiently activated even in mice receiving A^k^ bone marrow (Supplementary Fig. S2). We found that tumors were rejected only in chimeric mice that received A^b^ bone marrow cells (Fig. 3B, right). Thus, presentation of tumor antigen by bone marrow–derived myeloid cells is necessary for tumor rejection by effector CD4 T cells.

### *In Vivo* Interactions of Tumor-Specific CD4 T Cells Cause a Phenotypical and Functional Switch in the TAMs, from Tumor Promoting M2 to Inflammatory M1 Macrophages

**FIGURE 4:**
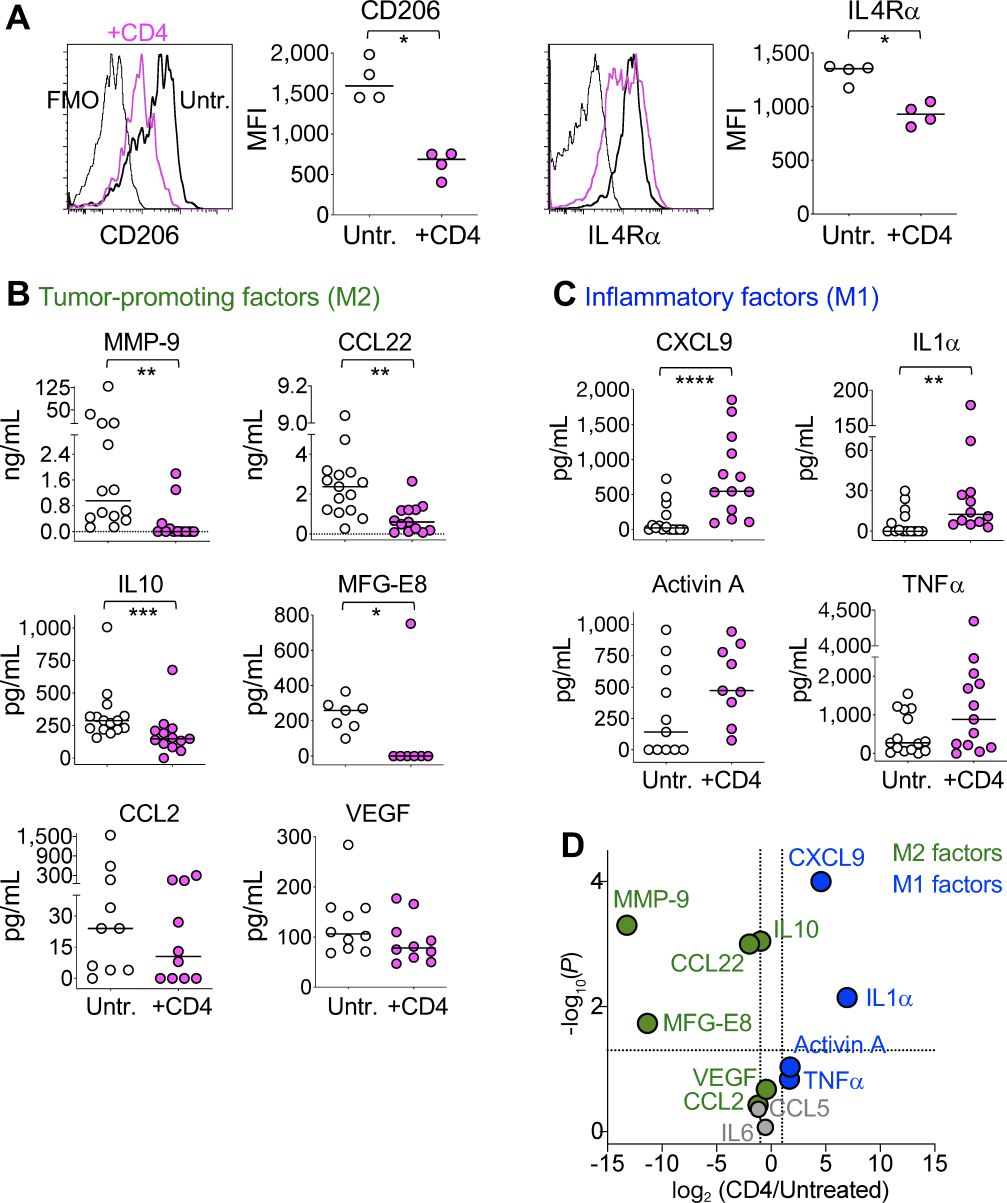
*In vivo* interactions of tumor-specific CD4 T cells cause a phenotypical and functional switch in the TAMs, from tumor-promoting M2 to inflammatory M1 macrophages. RAGKO mice were challenged with MB49 tumor. Five days later, half of the mice received anti-H-Y specific Marilyn CD4 T cells and 7 days later the TAMs (population I in Fig. 2B) from CD4 treated or untreated mice were compared. **A,** Representative histograms and mean fluorescent intensity (MFI) of CD206 (left) and IL4Rα (right) expression on TAMs coming from either untreated (white) or CD4-treated (pink) individual mice. Gray line in the histograms is unstained control. Data pooled from two independent experiments. **B–D,** Sorted TAMs coming from either untreated or CD4-treated mice (white vs. pink circles, respectively) were cultured overnight and tumor-promoting M2 factors (**B**) and inflammatory M1 factors (**C**) measured in the supernatants. **A–C**, Individual circles represent TAMs from individual mice. Horizontal lines are the medians in each experimental group. **D,** Volcano plot of combined data from **B**, **C**, and Supplementary Fig. S5 representing the ratio of the medians of each factor found in TAMS from CD4-treated versus untreated mice. Vertical discontinuous lines show 2-fold differences and horizontal discontinuous line shows *P* value of 0.05 by two-tailed Mann–Whitney test. Green and blue circles are M2 or M1 macrophage factors, respectively. Gray circles are factors not attributed to either group. **B–D**, Data compiled from three to nine independent experiments. **A–C**, *, *P* < 0.05; **, *P* < 0.01; ***, *P* < 0.001 using two-tailed Mann–Whitney test.

CD4 T cells modify the function of numerous other cells in the immune system. We hypothesized that, in similar way, effector CD4 T were able to modify the function of TAMs to induce tumor rejection. For this, we tested whether the *in vivo* interaction between Marilyn CD4 T cells and TAMs resulted in a phenotypical and/or functional change in the later ones. We compared TAMs (CD45^+^7AAD^−^CD11b^+^classII+Gr1^−^, population I in Fig. 2B) from untreated or CD4-treated mice 12 days after tumor challenge and 7 days after T-cell transfer, which is one day after the first CD4 T cells arrive at the tumor site (Supplementary Fig. S3). This timing enabled the *in vivo* TAM-CD4 T-cell interaction to occur for approximately 24 hours. Phenotypically, TAMs from CD4-treated mice showed significantly lower expression of Mannose receptor (CD206) and IL4Rα than TAMs from untreated mice (Fig. 4A), while other markers like CCR2, stayed unchanged (Supplementary Fig. S4A). To test for functional changes, we sorted the same TAMs from either untreated or Marilyn-treated mice, plate them overnight without further manipulation and measured the supernatants for various proteins characteristic of M2/tumor-promoting and M1/inflammatory macrophages. We found that TAMs from CD4-treated mice produced significantly less amount of the M2 products MMP-9, CCL22, IL10, and MFG-E8 (Fig. 4B), while producing significantly higher amount of M1 products, CXCL9 and IL1α (Fig. 4C) than TAMs from untreated mice. Although not statistically significant, TAMs from CD4-treated tumors produced less CCL2 (Fig. 4B) and CCL5 (Supplementary Fig. S4B) than TAMs from untreated mice, and more than double of the M1 factors Activin A and TNFα (Fig. 4C). TAMs products not affected by the presence of CD4 T cells were VEGF (Fig. 4B) and IL6 (Supplementary Fig. S4B). Altogether, this proteomic analysis showed that in presence of tumor specific CD4 T cells, the TAMs functionally switched from a tumor nurturing to a tumor-inflaming activity (Fig. 4D). These data show that tumor-specific CD4 T cells induce a phenotypical and functional change in TAMs *in vivo*, educating them not only to produce lower amounts of tumor-promoting factors, but also driving them to produce inflammatory factors that might assist in the CD4 T cell–mediated tumor rejection process.

### *In Vivo* CD4 Education of TAMs is Driven by an IFNγ Responsive Gene Signature

**FIGURE 5:**
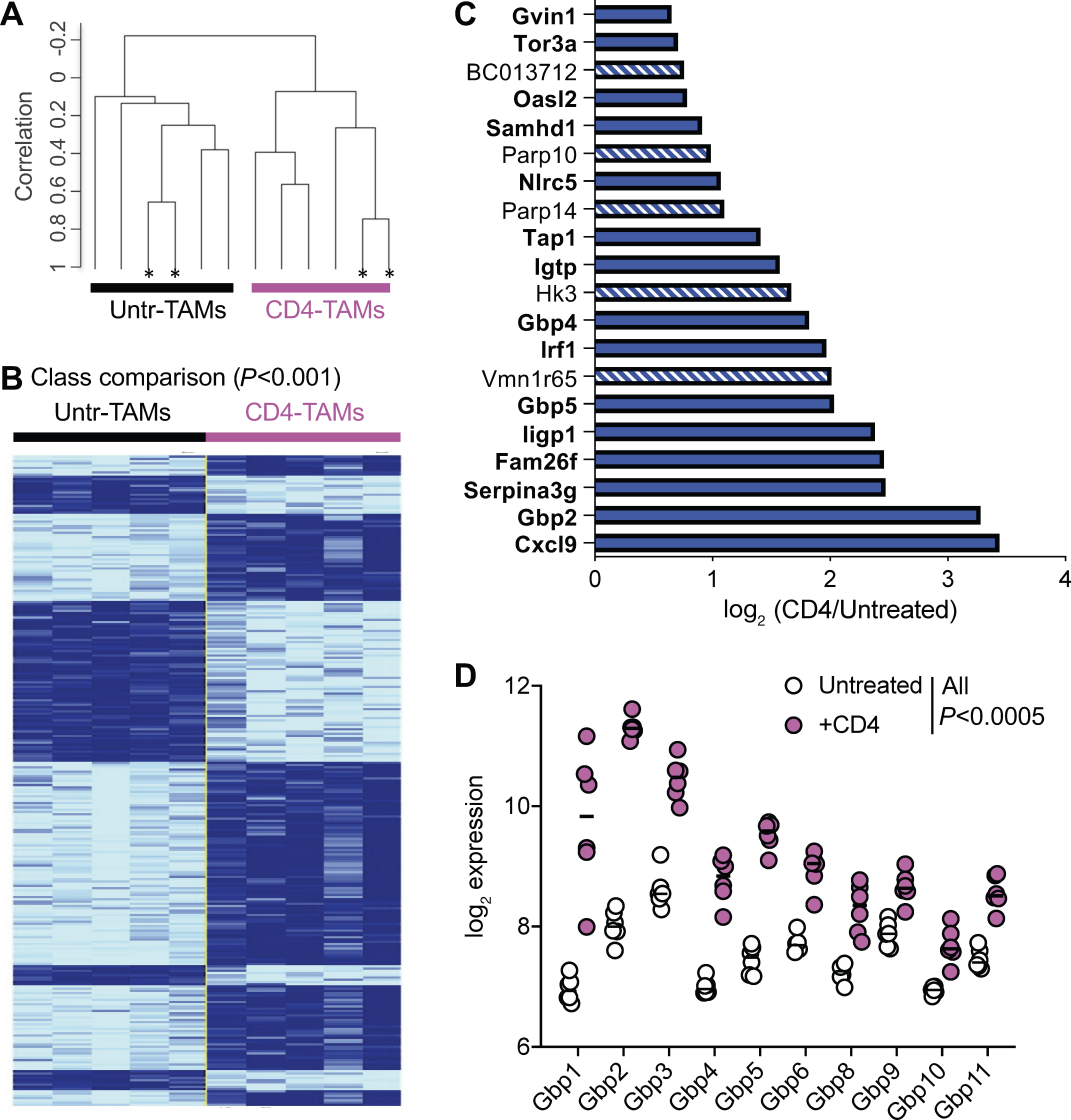
*In vivo* CD4 education of TAMs is driven by an IFNγ responsive gene signature. Sorted TAMs (population I in Fig. 2B) from either untreated (black) or CD4-treated (pink) mice were compared at the transcriptional level. Each group of TAMs consisted of five samples (four from individual mice and one from pooled mice), obtained in two individual experiments. **A,** Hierarchical clustering based on 18,893 genes that passed the filtering criteria. *, Technical replicates. **B,** Class comparison identifying 291 differentially expressed genes between the two groups of TAMs at *P* < 0.001. Technical duplicates were averaged into one sample. **C,** log_2_ of the expression ratio of the 20 genes most significantly differentially expressed (*P* < 2 × 10^−6^) between TAMs coming from CD4-treated versus untreated mice. Genes in bold and solid bars have been described to be downstream of IFNγ signaling. **D,** log_2_ expression of the *Gbp* family of genes in TAMs from CD4-treated or untreated mice, in pink circles and white circles, respectively. All *Gbp* with *P* < 0.0005 after Benjamini, Krieger, and Yekutieli correction for multiple comparisons.

To investigate the mechanism by which the CD4 T cells induce the phenotypical and functional switch in the TAMs, we compared the molecular signature of sorted CD11b^+^/class II+ cells from untreated versus CD4-treated tumors as in Fig. 4 (and population I in Fig. 2B). Unsupervised clustering analysis showed that 24 hours after *in vivo* interaction, the TAMs from CD4-treated mice were globally different to the TAMs from untreated mice as all the samples coming from CD4 treated mice clustered together and separated from the untreated mice samples (Fig. 5A). In addition, a supervised analysis using class comparison, unveiled 291 genes differentially expressed (*P* < 0.001) between CD4 treated and untreated TAMs (Fig. 5B). Of the 20 top genes whose expression changed the most significantly in presence of CD4 T cells, (*P* ≤ 2 × 10^−6^), 15 are known to be induced by IFNγ signaling (Fig. 5C, in bold). Of note, out of these 20 top genes, *Cxcl-9* showed the biggest increase in expression in TAMs from CD4-treated mice, in agreement with our previous findings at the protein level (Fig. 4C and D). Three of the 20 top genes belonged to the family of Guanylate-binding proteins (Gbp), a family of 11 genes that are important components of macrophage responses against bacterial and viral infections (30). Indeed, 10 of the 11 *Gbp* genes were upregulated in TAMs after *in vivo* interaction with the CD4 T cells (Fig. 5D). Thus, the *in vivo* CD4 T-cell education of TAMs seems to be characterized by a strong IFNγ gene signature, including a family of genes critical for antiviral and antibacterial responses.

### IFNγ Production by CD4 T Cells is Necessary for their Antitumor Effect

**FIGURE 6:**
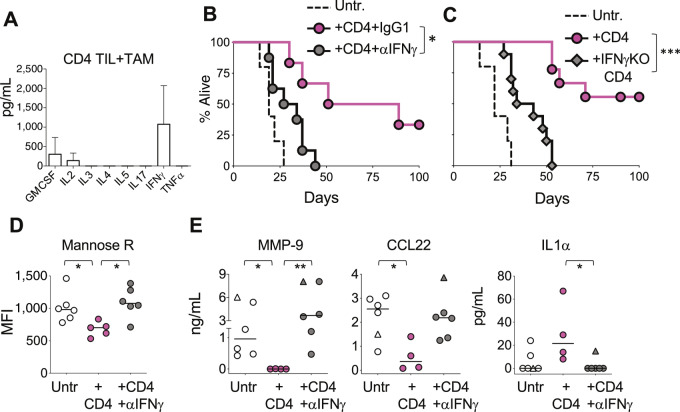
IFNγ production by CD4 T cells is necessary for their antitumor effect. **A,** Sorted anti-H-Y CD4 T Marilyn cells infiltrating the H-Y–expressing tumor MB49 were stimulated *in vitro* with TAMs sorted from untreated mice and cytokines were measured in the supernatants 24 hours later. Data show one of two experiments done. **B,** Survival of tumor-bearing mice was followed in three groups of mice; untreated (dashed line) and treated with Marilyn CD4 T cells in the presence of either anti-IFNγ blocking antibody (gray circles), or control rat IgG1 antibody (pink circles). Representative experiment from two done. **C,** As in **B**, this time comparing untreated mice (discontinuous line) with mice treated with either wild-type (pink circles) or IFNγ KO (gray diamonds) Marilyn CD4 T cells. **D–E,** TAMs from untreated (open circles), or CD4-treated mice receiving either control IgG1 antibody (pink circles) or anti-IFNγ blocking antibody (gray circles) were purified and their phenotype and function measured as in Fig. 4. **D,** Mean fluorescent intensity of CD206 on TAMs measured by flow. Data pooled from two independent experiments. **E,** Sorted TAMs were cultured overnight and proteins measured in the supernatants. Included are TAMs from untreated or CD4-treated RAG/IFNγRdKO mice (triangles). Data pooled from three independent experiments. **D–E**, Individual circles represent TAMs from individual mice. Horizontal lines are the medians in each experimental group. **B–C**, *, *P* < 0.05; ***, *P* < 0.001, using log-rank test. **D–E**, *, *P* < 0.05; **, *P* < 0.01, using Kruskal–Wallis with Dunn as post-test.

To identify the cytokine profile that effector Marilyn CD4 T cells produced after encountering the tumor antigen-presenting TAMs, we sorted effector Marilyn CD4 T cells from MB49 tumors (CD4 TILs) and stimulated them overnight with TAMs sorted from untreated MB49 tumors without external addition of antigen. The Marilyn CD4 T cells mostly produced IFNγ, GMCSF, and IL2 (Fig. 6A), which is very similar cytokine secretion profile to the one observed after the same sorted TILs were stimulated with αCD3 plus αCD28 antibodies (Supplementary Fig. S5). Thus, MB49 TAMs can present tumor antigens *in situ* to effector CD4 TILs, triggering mainly an IFNγ response.

To determine whether IFNγ plays a role in the CD4 T cell–mediated antitumor effect, we treated tumor-bearing mice with Marilyn CD4 T cells in the presence of either αIFNγ or control IgG1 antibodies. As seen in other models (31, 32), blocking IFNγ abrogated the CD4-mediated long-term antitumor effect (Fig. 6B). To test whether the tumor-specific CD4 T cells were the source of the crucial IFNγ, we treated tumor-bearing mice with IFNγ deficient Marilyn CD4 T cells and found that, similarly to the IFNγ blocking experiment, most of the antitumor effect was lost (Fig. 6C). In contrast, blocking TNFα which, together with IFNγ, have been shown necessary to reject mouse stablished tumors (32), had no effect (Supplementary Fig. S6). This is perhaps not surprising, because TAM stimulation did not induce TNFα production on Marilyn CD4 T cells (Fig. 6A). These data suggest that tumor antigen presentation by TAMs stimulate IFNγ production by CD4 T cells, which is necessary for the CD4-mediated antitumor effect.

Furthermore, and in agreement with the transcriptomic data shown in Fig. 5, IFNγ was responsible for most of the TAMs functional and phenotypical transformation observed in presence of CD4 T cells, as blocking this cytokine abrogated some of the changes shown in Fig. 4 (Fig. 6D and E). Specifically, the downregulation of Mannose receptor on TAMs was dependent on IFNγ (Fig. 6D), as well as the drop of the M2 factors MMP-9 and CCL22, and the increase of the M1 factor IL1α (Fig. 6E). Similar results were obtained by analyzing TAMs from IFNγRKO mice (Fig. 6E, triangles) where the TAMs cannot be directly stimulated by INFγ. Other CD4-mediated changes in TAMs, like IL4Rα, MFG-E8, IL10, and CXCL9, were not affected by IFNγ blockade (Supplementary Fig. S7).

CD40–CD40L interactions are important for CD4-mediated education process of other immune cells (19, 33). In addition, previous work has shown CD40 signaling to trigger an M1-like phenotype on macrophages in a model of chemically induced sarcomas (34). We consequently tested the possible role that CD40–CD40L interaction might have in our *in vivo* model of CD4-mediated TAMs education, by giving αCD40L blocking antibody to CD4-treated tumor-bearing mice. For our surprise, we found that in presence of αCD40L antibody, the CD4-mediated TAM functional switch was mostly unaffected (Supplementary Fig. S8), with only CCL22 reverting to levels found in TAMs from untreated mice. Altogether, these data show that IFNγ production by CD4 T cells upon antigen encounter on TAM in the tumor microenvironment is responsible for both the TAMs functional switch and the antitumor effect.

### Both IFNγ Signaling and Cognate Antigen Interaction on the Same TAM are Necessary for CD4-Mediated Antitumor Effect

**FIGURE 7:**
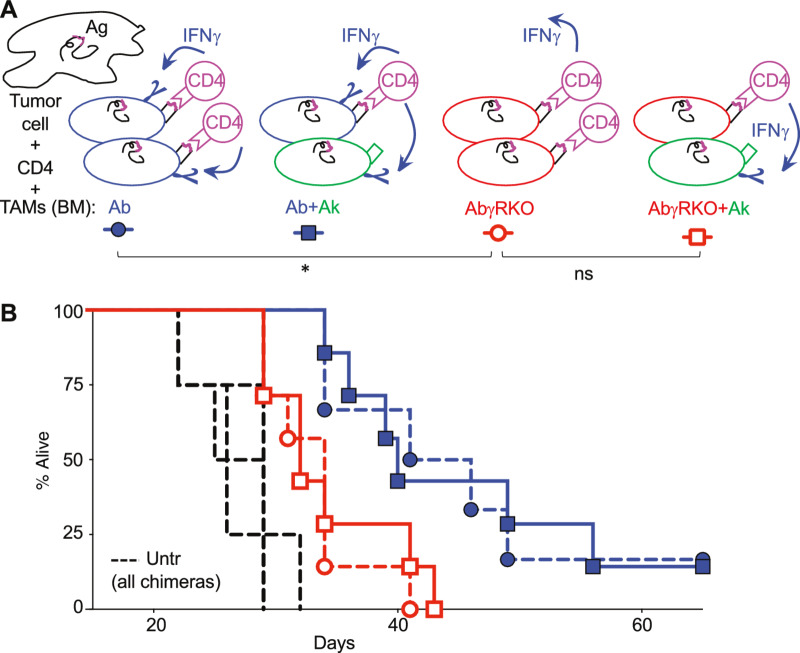
Both cognate interaction and IFNγ signaling on the same TAM are necessary for CD4-mediated antitumor effect. **A,** Representation of the tumor microenvironment conditions regarding antigen presentation and IFNγ signaling achieved by the four types of bone marrow chimera mice generated and whose results are shown in **B**. Irradiated B10.RAGKO mice were hosts of bone marrow (BM) cells from three different strains of mice (in blue, green, and red) or mixtures of them. **B,** Four groups of bone marrow chimeras were generated and can be divided in two subgroups; in the first subgroup (red symbols) the TAMs capable of presenting the H-Y antigen to Marilyn CD4 T cells (A^b^) are unable to receive IFNγ signals (γRKO; red cells in **A**) and are either alone (A^b^γRKO, red circles) or mixed with A^k^ TAMs (red squares). A^k^ TAMs cannot present H-Y antigen to the CD4 T cells but can receive IFNγ signals (green cells in **A**). In the second subgroup of chimeras (blue symbols), the TAMs capable of presenting the antigen can receive IFNγ signals (A^b^; blue cells in **A**) and are either alone (blue circles) or mixed with A^k^ TAMs (blue squares). Discontinuous lines represent untreated chimera mice. *, *P* < 0.05; ns, no significant, using log-rank test.

Data showed so far that for CD4-mediated tumor rejection is necessary antigen presentation by the TAMs and IFNγ signaling. We do not know, however, whether the TAMs need to directly receive the IFNγ signal and, if this were the case, whether both events need to occur on the same TAM or, on the contrary, they could happen on different TAMs of the tumor microenvironment. Both situations are possible as it has been shown that even though IFNγ is mostly secreted into the immunologic synapse, its effects are not completely restricted to the APC, but it can also affect non–antigen-presenting bystander cells (35). To answer these questions, and further investigate the mechanism of CD4-mediated tumor rejection, we used different bone marrow donor mice to obtain sets of chimera mice that allowed us to separate, at the cellular level, antigen presentation from IFNγ signaling occurrences. In the first set of chimeras, used as controls, antigen-presenting TAMs were able to receive IFNγ signal (Fig. 7A, blue symbols). The second set of chimeras was generated with either classII-Ab-γRKO (AbγRKO) bone marrow alone, or in combination with bone marrow from classII-Ak (Ak) mice. In AbγRKO chimera mice none of the TAMs could receive IFNγ signal (Fig. 7, red circles). In AbγRKO+Ak chimera mice, antigen presentation and IFNγ signaling were detached and only allowed to happen on different TAMs (Fig. 7, red square).

This experiment showed us three findings; first, simple bone marrow chimera mice, receiving bone marrow from either Ab or AbγRKO mice (Fig. 7, circles), showed that mice whose TAMs could not receive IFNγ signaling (AbγRKO, red circles) significantly lost the CD4 therapeutic effect seen in mice whose TAMs could receive IFNγ signal (Ab, blue circles). Second, mixed bone marrow chimera mice (Fig. 7, squares) showed that when antigen presentation was disassociated from IFNγ signaling, the CD4 therapeutic effect was lost (Fig. 7, compare red with blue squares). Third, the hindered antitumor effect observed when none of the TAMs could receive IFNγ signal (AbγRKO, red circle) is similar to the minimal effect observed when only the TAMs lacking CD4 cognate interaction could receive IFNγ signal (AbγRKO+Ak, red squares; Fig. 7B), suggesting that just IFNγ signaling on TAMs is not sufficient to induce tumor rejection. These data show that both cognate interaction and IFNγ signal on the same TAM are necessary for CD4-mediated antitumor effect.

## Discussion

We found that the interaction of effector CD4 T cells with TAM, a cell type previously extensively studied because of its role in promoting angiogenesis, metastasis, and tumor stem cell survival, can result on tumor rejection. We have identified the different steps necessary for this outcome to occur; TAMs need to be able to constitutively capture tumor antigens and present them to effector CD4 T cells at the tumor site. As a result of this stimulation, antigen-specific CD4 T cells produce IFNγ causing a phenotypical and functional switch in the TAMs, from tumor nurturing to an inflammatory M1-like phenotype. At the cellular level, we further discovered that the same TAM needs to receive both IFNγ signaling and cognate interaction with the CD4 T cells to obtain maximal antitumor response.

In contrast to a recent study where CD4 T cells with cytotoxic activity were found infiltrating human bladder cancers (36), we have previously shown that Marilyn CD4 T cells lack cytotoxic activity and that their antitumor effect does not rely on direct antigen recognition on tumor cells (1). Instead, CD4 T cells rely on direct cognate interaction on the same TAM that receives the IFNγ signal. Our study supports previous, and arguably understated, findings showing the crucial roles played by both IFNγ signaling on host cells (37) and antigen presentation by TAMs (38) for CD4-mediated tumor rejection. We also go further and clearly show that both stimuli (cognate antigen interaction and IFNγ signaling) need to be integrated on the same TAM for maximum antitumor effect. Consequently, these data suggest that IFNγ delivery at the tumor microenvironment alone, or in absence of CD4-TAM direct interaction, would not suffice for TAM reeducation and, most importantly, tumor clearance.

Although we initially showed the importance of CD4 T cells in tumor rejection in immunocompetent wild-type mice, we next moved to “immunocompromised” RAGKO mice as tumor bearing hosts to allow us to definitively demonstrate the significance of TAM and CD4s interactions without the distractions afforded by helped CD8 T cells, natural killer T cells or regulatory T cells (Treg). In addition, similar lymphopenic state has been shown to be necessary to increase the antitumor response success rate in patients with cancer before the adoptive transfer of tumor specific T cells (39). Furthermore, the use of MB49 tumor, spontaneously expressing H-Y as a neoantigen, allowed us to further elucidate the mechanisms of previously described clinical antitumor responses mediated by CD4 T cells specific to cancer neoantigens (2–4). The relevance of tumor MHC class II–restricted epitopes and their indirect presentation by DC and TAMs to trigger an antitumoral CD4 response, is additionally supported by previous findings describing the evolutionary pressure against MHC class II binding cancer mutations found in both mouse models (38), as well as in patients with cancer (40).

Molecularly, our data suggest that cognate interaction with IFNγ-producing CD4 T cells can disengage the TAMs from their tumor-associated genetic programs and divert them instead to a tumor-rejecting program. Notably in our model, the factor that CD4 T cells induced the highest increase of production by TAMs, both at the transcriptional and protein level, was CXCL9 which has been found to be a good predictor of the antitumor response obtained by checkpoint inhibitors (41, 42), suggesting that this therapeutic modality might be using similar mechanism of TAM education, by inhibiting negative signals given by antigen-presenting TAMs to CD4 T cells at the tumor site and increasing IFNγ production. Even though CXCL9 production is known to be dependent of IFNγ, we found that TAMs produced CXCL9 after *in vivo* IFNγ blockade (Supplementary Fig S7). It is possible that CXCL9 production by macrophages is particularly sensitive to small traces of IFNγ still present after anti-IFNγ blockade, because TAMs from CD4-treated IFNγRKO mice lacked CXCL9 production in the same type of experiment. We suggest that similar mechanism could explain how CD4 T cells caused the tumor microenvironment of a lymphoblastic lymphoma to remodel when the tumor oncogene was inactivated, inducing tumor regression (43).

Tumor rejection, however, is not the universal outcome of CD4/TAM interactions. For example, CD4 T cells of the “Th 2” type have been shown to enhance the frequency of lung metastases of mammary tumors through their interactions with TAMs (44), and, in a clinical setting, it has been found that the ratio of Th2 over Th1 cells infiltrating a tumor correlates with poor prognosis of patients with pancreatic cancer (45). Likewise, CD4 Tregs present at the tumor site could inhibit the antitumor effect of both CD4 and CD8 effector cells through interaction with antigen-presenting TAMs. Therefore, to achieve tumor clearance is imperative the presence of tumor specific CD4 T cells with Th1 effector function that can quantitatively and functionally overpower other potential CD4 T cells with detrimental functions such as Th2 and Tregs.

These combined data suggest two major corollaries. First, CD4 education of TAMs can occur in tumors originating in different tissues, and in both spontaneous and transplantable tumor models, suggesting that it is a widespread phenomenon. Second, the predominant effector type of CD4 T cells at the tumor site is relevant for cancer patient survival. Therefore, to use CD4 T cells as antitumor treatment it would be necessary to ensure that the right effector type of CD4 T cell reaches the tumor site. Thus, appropriately primed CD4 T cells may be useful for both stand-alone therapies as shown in a melanoma clinical trial (46) and as multi-faceted tumor immunotherapy. For example, because the tumor stroma can induce the tolerization of tumor specific CD8 T cells when these cells are given as a standing alone therapy, the addition of CD4 T cells has been shown to alter the immunosuppressive tumor microenvironment, preventing the CD8 T cells tolerization and enhancing their antitumor effects (47). In addition, because the tumor stroma supports cancer cell drug resistance (7, 48), our data suggest that CD4-based immunotherapy could act synergistically with chemoradiotherapy and radiotherapy by weakening the supporting role of TAMs and thus making the cancer cells more susceptible to the therapeutic agents. Furthermore, once the molecular mechanism of TAM phenotype switching is known, it may be possible to use small molecules and/or antibodies to manipulate the switch.

The initially surprising phenotype of the TAMs found in our model, with the combined expression of the macrophage and DC markers, F4/80 and CD11c, has previously been found in tumor-infiltrating myeloid cells (25), and also under inflammatory conditions in many peripheral tissues. For example, in the lung after intratracheal fungal infection (26), in the central nervous system during the onset of experimental autoimmune encephalomyelitis (29), and in the dermis after Leishmania major infection of the footpad (27). Such TAM-like cells have also been described under steady-state conditions surrounding the pancreatic islets and presenting pancreatic antigen to CD4 T cells (28). Depending on the study, these F4/80^+^/CD11c^+^ cells have been called either monocyte-derived DC (moDC; ref. 49), or tissue-resident macrophages (50), but the consensus supported, among other things, by their expression of CD64 (51), seems to be that they derive from blood monocytes, which are recruited to the tissues in a CCR2-dependent manner, where they differentiate into tissue APCs (51). These studies suggest that tissue macrophages, expressing CD11c and other costimulatory molecules, might play an important role not only in cancer, but also during immune responses in different tissues.

Therefore, we suggest that understanding the education of tissue resident macrophages/DC by effector CD4 T cells is not only relevant for tumor immunotherapy but also for other tissues undergoing immune responses, and, perhaps, for tissue homeostasis. For instance, in obesity and type 2 diabetes, M2-like macrophages keep the adipocytes responding to insulin (52), and Th2 CD4 cells seem to protect from insulin resistance in obesity models, whereas Th1 CD4 cells drive diabetes (53). In addition, in mouse models of atherosclerosis, the disease seems to be driven by IFNγ-producing CD4 T cells upon antigen recognition on DC/macrophages localized in the arterial wall (54). Likewise, in experimental autoimmune encephalomyelitis the pathogenic inflammation seems to be caused by GM-CSF producing CD4 T cells upon neuroantigen recognition on moDC/macrophages associated with vessels of the central nervous system (55).

Together with our data, these studies suggest that CD4 T cells recognizing antigens on tissue-resident APCs can be important modifiers of the inflammatory processes that can drive (or protect from) not only cancer but also other types of diseases, accentuating the importance of studying CD4 T cell–TAM interactions to harness them for future clinical treatments.

## Supplementary Material

Supplementary Figures S1-S7 Fig S1 shows that sorted CD4 T cells used in Figure 2 lack APC
Fig S2 shows that CD4 T cells in all chimeric mice shown in Figure 3 were primed by the immunizations
Fig S3 shows that CD4 T cells first arrive at the tumor site around day 6
Fig S4 shows the proteins secreted by TAMs that are not changed by in vivo interaction with tumor specific CD4 T cells
Fig S5 shows the cytokines produced by sorted CD4 TILs after anti-CD3 and anti-CD28 in vitro stimulation
Fig S6 shows that in vivo blockade of TNF-alpha does not inhibit the CD4 mediated anti-tumor effect
Fig S7 shows the factors characteristic of the TAMs re-education by CD4 T cells that are independent of IFN-gamma
Fig S8 shows that the CD4 mediated in vivo phenotypic switch of TAMs is not dependent on CD40L

Supplementary Experimental Procedures Supplementary Materials and Methods
