# Supplementary material for: Neoantigen Presentation and IFNγ Signaling on the Same Tumor-associated Macrophage are Necessary for CD4 T Cell–mediated Antitumor Activity in Mice
Source: Cancer Res Commun. 2022 May 9;2(5):316–29. doi: 10.1158/2767-9764.CRC-22-0052 (PMC9321644; doi:10.1158/2767-9764.CRC-22-0052)
Supplement: Supplementary Figures S1-S7 — Fig S1 shows that sorted CD4 T cells used in Figure 2 lack APC Fig S2 shows that CD4 T cells in all chimeric mice shown in Figure 3 were primed by the immunizations Fig S3 shows that CD4 T cells first arrive at the tumor site around day 6 Fig S4 shows the proteins secreted by TAMs that are not changed by in vivo interaction with tumor specific CD4 T cells Fig S5 shows the cytokines produced by sorted CD4 TILs after anti-CD3 and anti-CD28 in vitro stimulation Fig S6 shows that in vivo blockade of TNF-alpha does not inhibit the CD4 mediated anti-tumor effect Fig S7 shows the factors characteristic of the TAMs re-education by CD4 T cells that are independent of IFN-gamma Fig S8 shows that the CD4 mediated in vivo phenotypic switch of TAMs is not dependent on CD40L [file crc-22-0052-s01.pdf]

Supplementary Figure S1

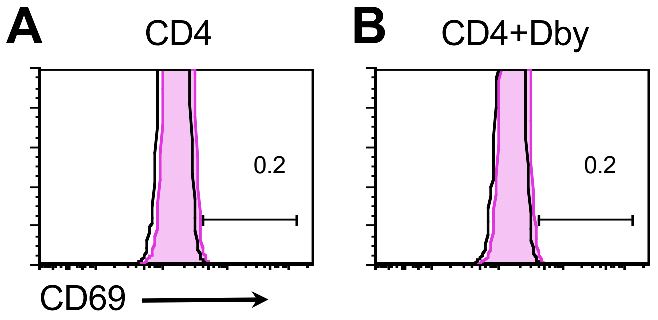

Supplementary Figure S1. Sorted CD4 T cells used in Fig. 2C lack Antigen Presenting Cells (APC) contaminants. CD69 expression on naïve sorted anti-H-Y Marilyn CD4 T cells in pink and A1M anti-H-Y CD4 T cells (which recognize H-Y presented by MHC allele A^k) in open black line, after 24h of culture in either **A,** medium alone, or **B,** medium plus 5 mM of the Dby (H-Y) peptide. Marilyn cells were analyzed by gating on CD4+/TCR+/Vβ6+ cells and A1M cells by gating on CD4+/TCR+/Vβ6-. The lack of CD69 upregulation by Marilyn cells in presence of Dby shows the lack of APC in the CD4 sorted cells. A representative experiment of three is shown.

Supplementary Figure S2

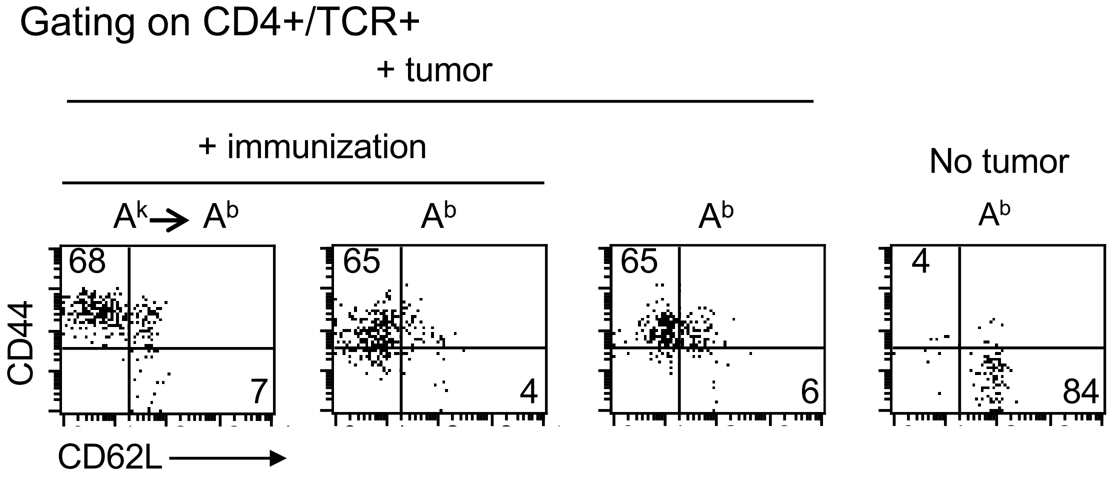

Supplementary Figure S2. Marilyn CD4 T cells are activated after immunization with A^b male splenocytes in the bone marrow chimera mice shown in Fig. 3. As described in Fig. 3, mice received A^k bone marrow cells, Marilyn CD4 T cells, and were immunized four times with A^b male splenocytes. Five days after the last immunization, their spleens were analyzed by flow cytometry for the activation markers (CD44-high/CD62L-low) gating on CD4+/TCR+ cells. As controls, A^b mice that received Marilyn CD4 T cells and were left naïve (no tumor), or received tumor cells but not immunization, or received both. A representative mouse of three is shown.

Supplementary Figure S3

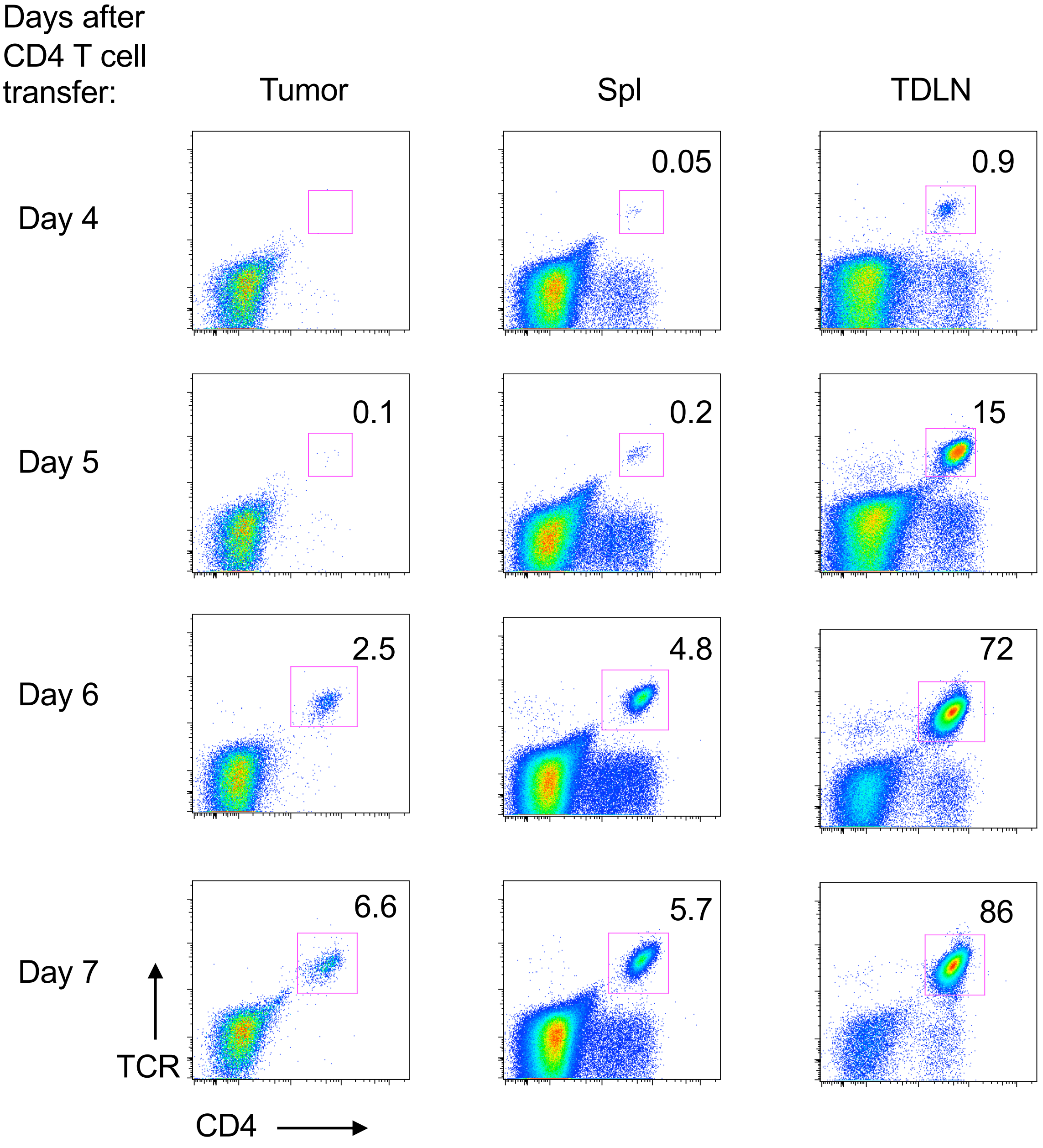

Supplementary Figure S3. Kinetics of CD4 T cell arrival at the tumor site compared to spleen and tumor draining lymph node. Mice were injected s.c. with H-Y expressing MB49 tumor cells and naive Marilyn anti-H-Y CD4 T cells were transferred five days later. Four, five, six and seven days after T cell transfer, mice were euthanized and tumor, spleen (spl), and tumor draining lymph nodes (TDLN) were analyzed by flow cytometry. Data are gated on CD45+/7AAD- cells, and numbers represent the percentage of CD4+/TCR+ within the gated population. Representative mice from two independent experiments

Supplementary Figure S4

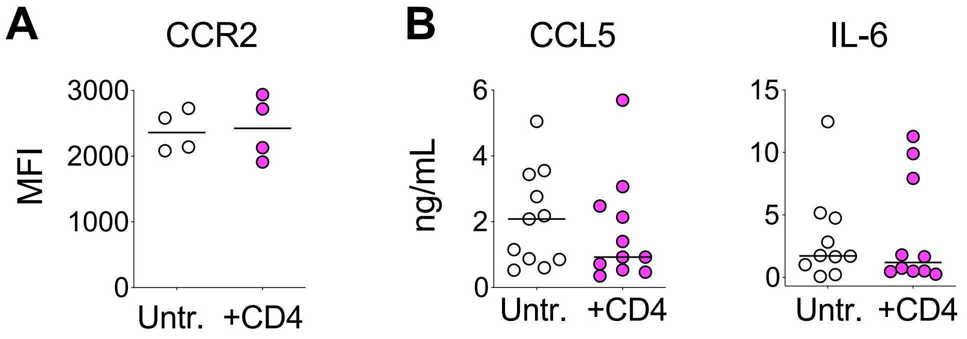

Supplementary Figure S4. Products whose expression do not change in TAMs after in vivo interaction with tumor specific CD4 T cells. **A-B,** TAMs from untreated (open circles), or CD4-treated mice receiving (pink circles) were purified and their phenotype and function measured as in Fig. 4. **A,** Mean fluorescent intensity of CCR2 on TAMs measured by flow. Data pooled from the same two independent experiments shown in Fig. 4A. **B**, Sorted TAMs were cultured overnight and proteins measured in the supernatants. Data pooled from three to nine individual experiments. **A-B**, Individual circles represent TAMs from individual mice. Horizontal lines are the medians in each experimental group.

Supplementary Figure S5

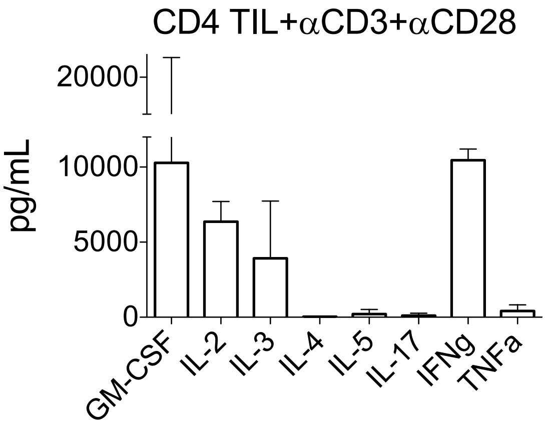

Supplementary Figure S5. IFN-γ production by sorted CD4 Tumor Infiltrating Lymphocytes (TIL) after stimulation with αCD3 plus αCD28. Cytokines were measured in the supernatants 24 h. later. Data show one out of two experiments done.

Supplementary Figure S6

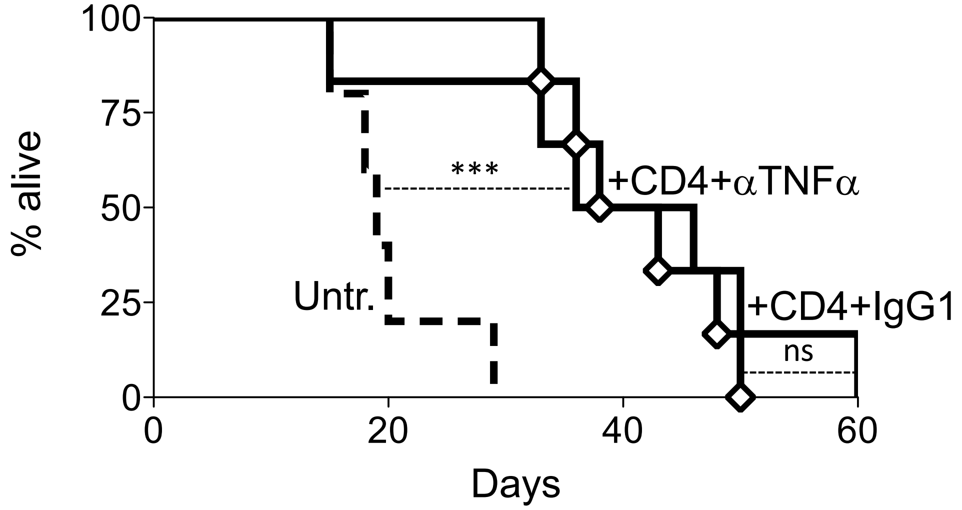

Supplementary Figure S6. TNF-α is not necessary for CD4 mediated anti-tumor effect. Survival of tumor bearing mice was followed in three groups of mice injected with MB49 tumor. Mice were left untreated (discontinuous line), or treated five days later with Marilyn CD4 T cells in the presence of either anti-TNF-α blocking antibody (open diamonds), or rat IgG1 isotype control antibody (continuous line). ***, $p<0.001$; ns, not significant using Logrank test.

Supplementary Figure S7

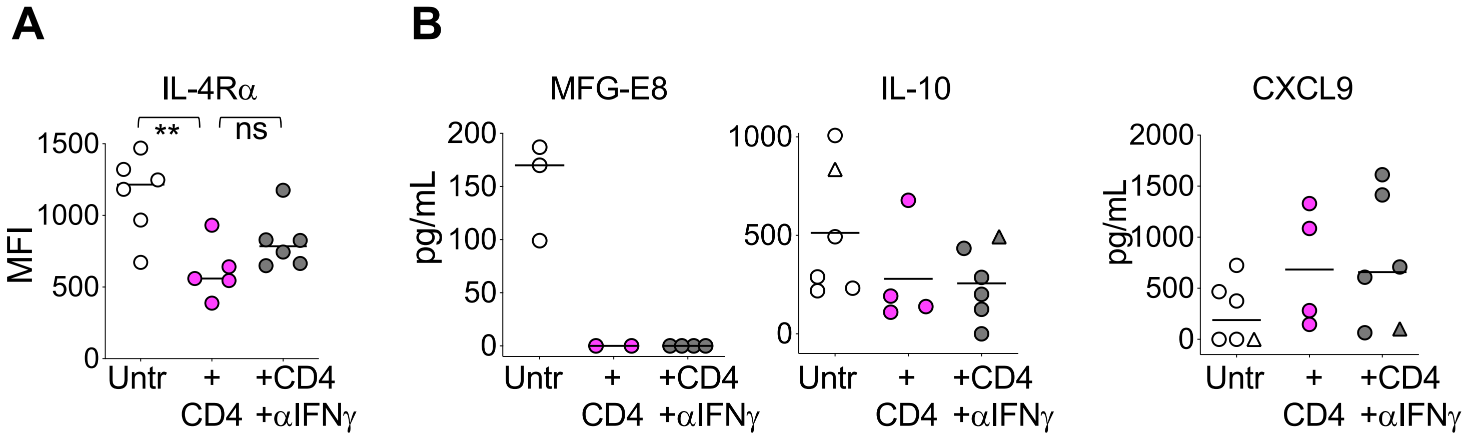

Supplementary Figure S7. CD4 mediated changes on TAMs that are IFN-γ independent. **A-B,** TAMs from untreated (open circles), or CD4-treated mice receiving either control IgG1 antibody (pink circles) or anti-IFN-γ blocking antibody (grey circles) were purified and their phenotype and function measured as in Fig. 4. **A,** Mean fluorescent intensity of IL-4Rα on TAMs measured by flow. Data pooled from two independent experiments. **B**, Sorted TAMs were cultured overnight and proteins measured in the supernatants. Included are TAMs from untreated or CD4-treated RAG/IFNγRdKO mice (triangles). Data pooled from three independent experiments. **A-B**, Individual circles represent TAMs from individual mice. Horizontal lines are the medians in each experimental group. **, $p<0.01$, using Kruskal-Wallis with Dunns as post-test.

Supplementary Figure S8

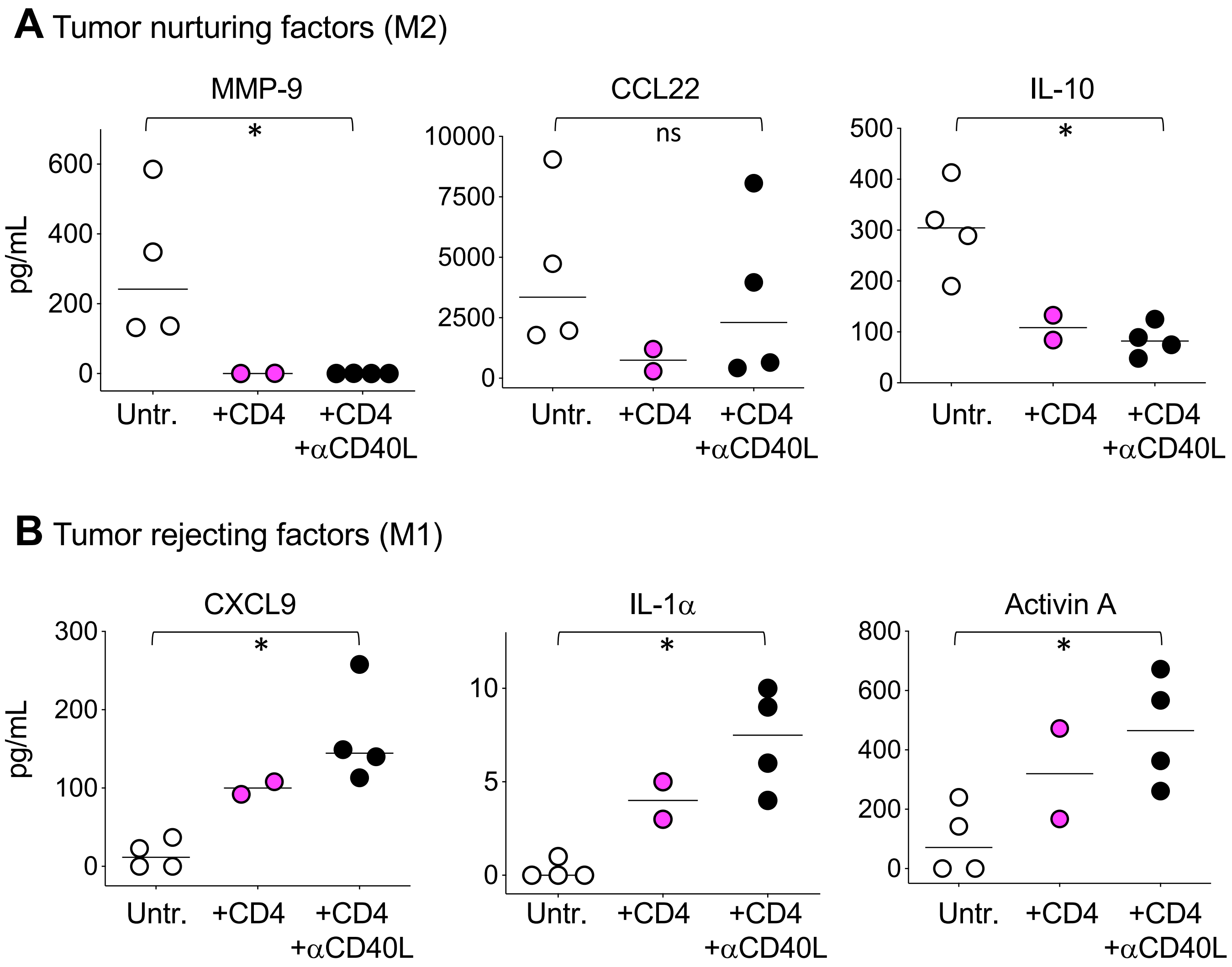

Supplementary Figure S8. CD4 mediated changes on TAMs do not require CD40-CD40L interaction. TAMs were sorted and analyzed in the same way as in Fig. 4 with the addition of either αCD40L antibody (black circles), or control hamster antibody (pink circles) to MB49 tumor bearing and Marilyn CD4 treated mice. Open circles represent TAMs sorted from untreated mice. Antibodies (800 μg) were given at days two and five after T cell transfer. Horizontal bars represent the median for each factor in each experimental group. Data pooled from two independent experiments. *, $p<0.05$ using two tailed Mann-Whitney test. ns, no significant.
