# Supplementary material for: Neoantigen Presentation and IFNγ Signaling on the Same Tumor-associated Macrophage are Necessary for CD4 T Cell–mediated Antitumor Activity in Mice
Source: Cancer Res Commun. 2022 May 9;2(5):316–29. doi: 10.1158/2767-9764.CRC-22-0052 (PMC9321644; doi:10.1158/2767-9764.CRC-22-0052)
Supplement: Supplementary Experimental Procedures — Supplementary Materials and Methods [file crc-22-0052-s02.docx]

**Supplemental Experimental Procedures**

**Antibodies used for flow cytometry**

From BD Pharmingen, CD11b (M1/70), CD62L (MEL-14), CD4 (RM4-5), Gr-1 (RB6-8C5), CD8α (53-6.7), CD40 (HM40-3), CD45.2 (104), MHC Class II A^b^ (7-16.17 and AF6-120.1), TCR Vβ6 (RR4-7), MHC Class II A^k^ (10-3.6), CD124 (mIL4R-M1), CD11c (HL3), TCRαβ (H57-597), CD80 (16-10A1), and CD86 (GL-1). From eBioscience, CD44 (IM7), CD69 (H1.2F3), and CD103 (2E7). From Biolegend, CD206 (MR5D3), and PD-L1 (10F.9G2). From Caltag, F4/80 (BM8). From R&D Systems, CCR2 (475301).
